# Characterization of writing in patients with the semantic variant of Primary Progressive Aphasia

**DOI:** 10.1002/alz.092525

**Published:** 2025-01-03

**Authors:** Thais Helena Machado, Rafaela Souza Pereira, Aline Carvalho Campanha, Caroline Martins de Araujo, Maria Teresa Carthery‐Goulart, Elisa de Paula França Resende, Leonardo Cruz de Souza, Paulo Caramelli

**Affiliations:** ^1^ Universidade Federal de Minas Gerais, Belo Horizonte, Minas Gerais Brazil; ^2^ UFMG, Belo Horizonte‐MG, Minas Gerais Brazil; ^3^ Federal University of Minas Gerais, Belo Horizonte, Minas Gerais Brazil; ^4^ Universidade Federal de Minas Gerais, Belo Horizonte‐MG, Minas Gerais Brazil; ^5^ UFABC, São Bernardo do Campo, São Paulo Brazil

## Abstract

**Background:**

Language impairment manifestations in patients with the semantic variant of primary progressive aphasia (S‐PPA) include surface dysgraphia. Word and pseudoword dictation helps characterize errors in these patients’ writing to better conduct their therapeutic strategies. Few studies have addressed written language in patients with dementia, and even fewer have done so in PPA. The objective was to analyze, classify, and quantify the types of writing errors in adults with S‐PPA using word and pseudoword dictation.

**Methods:**

This cross‐sectional study was conducted in a Cognitive and Behavioral Neurology Outpatient Clinic. Its project was approved by the Research Ethics Committee. The research analyzed 19 patients with S‐PPA and 30 healthy individuals using a word and pseudoword dictation protocol. The two groups were matched for age, sex, and education level. The inclusion criteria for the control group were subjects whose Mini‐Mental State Examination score was appropriate to their age and education level, without any neurological and/or psychiatric diseases, and not taking medications that interfere with cognition. For the patients’ group, they were only required to be medically diagnosed with S‐PPA. Descriptive statistical analysis was performed, showing data frequency.

**Results:**

The S‐PPA group performed worse than controls on the word and pseudoword writing task. Both groups performed worse in irregular words and grapheme substitution.

**Conclusion:**

Patients with S‐PPA performed worse in word and pseudoword writing than controls, most often in grapheme substitution and irregular words, which can be explained by the frequent occurrence of surface dysgraphia present in S‐PPA, which hinders their access to the lexical route.

